# Effects of acupuncture and computer-assisted cognitive training for post-stroke attention deficits: study protocol for a randomized controlled trial

**DOI:** 10.1186/s13063-015-1054-x

**Published:** 2015-12-02

**Authors:** Jia Huang, Michael A. McCaskey, Shanli Yang, Haicheng Ye, Jing Tao, Cai Jiang, Corina Schuster-Amft, Christian Balzer, Thierry Ettlin, Wilfried Schupp, Hartwig Kulke, Lidian Chen

**Affiliations:** College of Rehabilitation Medicine, Fujian University of Traditional Chinese Medicine, No.1 Huatuo Road, Minhou District, Fuzhou, 350122 Fujian Province China; Fujian University of Traditional Chinese Medicine Affiliated Rehabilitation Hospital, No. 282 Wusi Road, Fuzhou, 350003 Fujian Province China; Research Department, Reha Rheinfelden, Salinenstrasse 98, 4310 Rheinfelden, Switzerland; m&i-Fachklinik Herzogenaurach, In der Reuth 1, 91074 Herzogenaurach, Germany

**Keywords:** Stroke, Computer-assisted cognitive training, Acupuncture, Attention deficits

## Abstract

**Background:**

A majority of stroke survivors present with cognitive impairments. Attention disturbance, which leads to impaired concentration and overall reduced cognitive functions, is strongly associated with stroke. The clinical efficacy of acupuncture with Baihui (GV20) and Shenting (GV24) as well as computer-assisted cognitive training in stroke and post-stroke cognitive impairment have both been demonstrated in previous studies. To date, no systematic comparison of these exists and the potential beneficial effects of a combined application are yet to be examined. The main objective of this pilot study is to evaluate the effects of computer-assisted cognitive training compared to acupuncture on the outcomes of attention assessments. The second objective is to test the effects of a combined cognitive intervention that incorporates computer-assisted cognitive training and acupuncture (ACoTrain).

**Methods/Design:**

An international multicentre, single-blinded, randomised controlled pilot trial will be conducted. In a 1:1:1 ratio, 60 inpatients with post-stroke cognitive dysfunction will be randomly allocated into either the acupuncture group, the computer-assisted cognitive training group, or the ACoTrain group in addition to their individual rehabilitation programme. The intervention period of this pilot trial will last 4 weeks (30 minutes per day, 5 days per week, Monday to Friday). The primary outcome is the test battery for attentional performance. The secondary outcomes include the Trail Making Test, Test des Deux Barrages, National Institute of Health Stroke Scale, and Modified Barthel Index for assessment of daily life competence, and the EuroQol Questionnaire for health-related quality of life.

**Discussion:**

This trial mainly focuses on evaluating the effects of computer-assisted cognitive training compared to acupuncture on the outcomes of attention assessments. The results of this pilot trial are expected to provide new insights on how Eastern and Western medicine can complement one another and improve the treatment of cognitive impairments in early stroke rehabilitation. Including patients with different cultural backgrounds allows a more generalisable interpretation of the results but also poses risks of performance bias. Using standardised and well-described assessments, validated for each region, is pivotal to allow pooling of the data.

**Trial registration:**

Clinical Trails.gov ID: NCT02324959 (8 December 2014)

## Background

Cognitive impairment is a condition characterised by mental deficits. Cognitive deficits occur in more than half of stroke survivors and are deemed to be more important determinants of health- and quality of life-related outcomes than physical disability [1, 2]. The most common type of cognitive deficit is impaired attention, with a point prevalence between 46 % and 92 % reported in acute stroke survivors [3]. These deficits along with difficulties to concentrate and continued decline in cognitive functions severely affect quality of life [4–7].

Attention is closely related to and involved in most cognitive functions, such as most processes of perception, memory [8], planning and acting [8], speech perception [9], spatial orienting [10], and social problem-solving [11]. Accordingly, attention can be considered a basic competence necessary for most practical and intellectual performances [12]. Consequently, it cannot be isolated, whether conceptually or functionally, from other cognitive functions. It has further been shown that these deficits impair certain motor abilities and inhibit motor learning [13]. Impairments of attention are a major problem for people with stroke and affect rehabilitation outcome [14]. A systematic review on cognitive training for post-stroke attention deficits, which included six randomised controlled trials (RCTs) with 223 participants [14], suggested that there may be a short-term effect on attention abilities. However, due to the methodological limitations of the reported studies, there was insufficient evidence to support or refute the persisting effects of cognitive rehabilitation on attention or on functional outcomes.

Acupuncture is one of the main modalities of treatment in Traditional Chinese Medicine (TCM) and has a practical history of more than 2000 years in China [15]. A recent meta-analysis [16] was conducted to evaluate the efficacy of acupuncture on cognitive impairment (function) after stroke. Twenty-one trials with a total of 1421 patients were included. The cumulative findings suggest that acupuncture has positive effects on cognitive function after stroke. According to TCM theory of acupuncture, the Du meridian plays an important role in treating attention problems [17]. Baihui (GV20) and Shenting (GV24) are two main acupoints from the Du meridian which are believed to activate the spirit. Previous studies with experimentally induced cerebral ischemia injuries in rodents [18, 19] showed improved learning and memory abilities after 4 weeks (five times per week, 30 minutes per time) of Baihui (GV20) and Shenting (GV24) acupuncture treatment. However, the existing literature of the effects of acupuncture on cognitive impairment after stroke describes general cognitive functions and do not provide sufficient evidence as to how acupuncture could improve attention deficits after stroke.

The aims of our study are: (1) to observe the effect of acupuncture combined with RehaCom training on cognitive dysfunction (in particular attention disturbances) after stroke, and (2) to test efficacy compared to conventional treatment combined with acupuncture or conventional treatment combined with RehaCom training.

## Methods

### Study design and setting

**Fig. 1:**
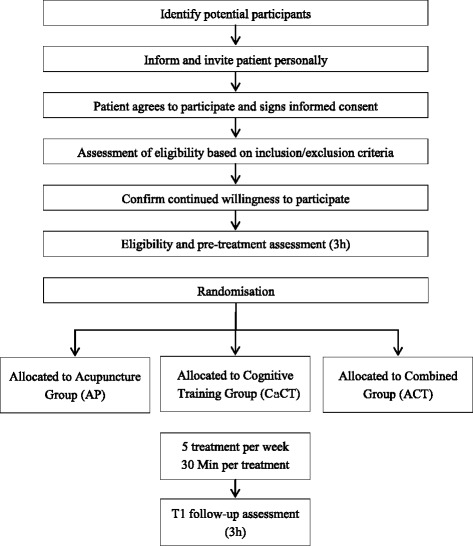
Flow diagram of study design including the process of recruitment, allocation and intervention, and assessment

The study is designed as an international multicentre study with randomised, controlled group comparators and blinded assessors. The study will be an explorative pilot study with three parallel treatment groups: computer-assisted cognitive training (CaCT), acupuncture (AP), and intervention that incorporates CaCT and AP (ACoTrain, ACT). Three neurological rehabilitation clinics from three different countries will be involved in recruitment, treatments and assessments. Each clinic will aim to recruit 20 inpatients currently recovering from their clinically first-ever stroke. Attention performance will be assessed as the primary study outcome in all groups before and after a 4-week intervention. The multicentric body of trial centres consists of specialised neurological rehabilitation centres from China (Fujian University of Traditional Chinese Medicine, A), Switzerland (Reha Rheinfelden, B), and Germany (m&i Fachklinik Herzogenaurach, C). Fig. 1 illustrates the flow chart of patient allocation and study design.

### Sample size

The international setting is thought to increase the generalisability of the study results, as the study sample will include patients with different cultural background. However, it is also expected that high heterogeneity regarding affected cortical areas and functional ability within the study groups of stroke patients will occur. The number of participants will therefore not suffice to conduct a full-scale study but are thought to provide a basis for estimation of the effects and planning of a larger follow-up study, if proven feasible.

The aim is to recruit 20 patients per clinic (N_total_ = 60). The results will be used to compute sample size and conduct a power calculation to plan the full-scale follow-up study.

### Participant and recruitment

The participants will be recruited from three inpatient stroke rehabilitation units from (1) Fujian University of Traditional Chinese Medicine Subsidiary Rehabilitation Hospital; (2) m&i-Fachklinik Herzogenaurach; and (3) Reha Rheinfelden. Patients will be identified and recruited via the clinic’s database according to diagnosis and medical referral. Respective medical personnel will be asked to invite patients from the population under investigation. Patients who express a general interest in taking part in the study will meet with one of the study personnel to receive all necessary information in oral and written form. After a 24-hour consideration period, the patient will be reapproached by the investigator to resolve any uncertainties and to be invited to sign informed consent after an assessment of eligibility criteria. After baseline assessment, patients who meet the inclusion criteria will be randomly allocated to one of the three study groups. A CONSORT diagram of participant recruitment is shown in Table 1.

**Table 1:** Trial processes chart

Study periods	Admission	1^st^ *c*ontact	2^nd^ *c*ontact	Eligibility	Assessment	Treatment	Follow-up
Visit Duration	Week 1	Week 1	Week 1	Week 1	Week 1	Week 2–5	Week 6
		10 min	10 min	1 hour	3 hours	(4 × 5 × 30 min)	3 hours
Time	−7d	−6d	−5d	−4d	−4-0d	−2d	27d
Admission	x						
Medical examination	x						
Patient information		x			x		
Consultation			x				
Informed consent				x			
Demographics				x			
Medical history				x			
In-/exclusion Criteria				x			
Randomisation						x	
Administer study						x	
Primary variables					x		x
Secondary variables					x		x
Concomitant therapy						x	
Adverse events						x	x

### Inclusion criteria

In order to properly understand and apply the training methods and the content of the study, the patients are required to have some level of cognition. To assess the minimum level required, the Montreal Cognitive Assessment (MoCA) [20] will be applied at baseline (BL). Patients eligible for the trial must comply with all of the following criteria prior to randomisation: (1) clinically first-ever stroke, diagnosis confirmed by CT or MRI; (2) aged between 18 and 80 years old; (3) clinically confirmed attention deficits (>1SD below mean of age-matched norm in at least one subtest of the Test for Attentional Performance, TAP); (4) inclusion within 6 months after stroke onset; (5) conscious, stable physical condition; and (6) given oral and written consent confirmed by the signature of the patient.

### Exclusion criteria

Patients who are positive to one or more of the following criteria will have to be excluded: (1) self-reported severe visual problems (inability to recognise illustrations on MoCA); (2) hearing problems (TAP discrimination of tones >1SD of aged-matched norm); (3) notable deficiencies in MoCA submodalities on visuospatial and attention assessments, neglect or hemianopia <2 points on visuospatial assessments, failed attention test (read list of letters and tap on every *A*), inability to concentrate throughout the MoCA assessment, inability to execute a standardised three-step instruction: “*Please take this piece of paper, fold it twice to fit it in this standardized envelope and hand it back to me*.”; (5) speech comprehension deficits: <9 points on the short aphasia test (KAP); (6) seizures; (7) pre-existing psychological disorders prior to stroke; (8) severe medical condition (e.g. critical diabetes with hyper- or hypoglycemia); (9) pregnancy (in doubt, a pregnancy test will be required); (10) self-reported, family-reported or neuropsychological assessment proves cognitive decline in history before the onset of stroke.

### Randomization and blinding

For the randomisation, restricted randomisation with random block sizes between 3 and 9 (exact size to be determined by an external institution) will be used. This will reduce opportunities for bias and confounding and increases the probability to meet the assumptions of statistical analysis (treatment groups are random samples of population) [21]. Each centre will receive a computer-generated randomisation list (MATLAB 2007b, Mathworks Inc., Natick, MA, USA) from a third-party partner institution, which is elsewise not involved in the study. Sequentially numbered opaque and sealed envelopes will be distributed to each trial centre by a third-party employer, who is not part of the investigating team. The treating therapist will inform the participants after baseline assessment immediately before the first treatment. The assessor remains uninformed about group allocation. Patients will be informed and reminded not to mention the study treatment in front of the assessors.

### Ethical issues

This study will be conducted in compliance with study protocol and the current version of the Declaration of Helsinki. All participants will be fully informed about the trial, and will sign the written informed consent form prior to participation. The trial has been approved by the ethics committee of the Rehabilitation Hospital affiliated to Fujian University of Traditional Chinese Medicine (Approval No. 2013KY-004-02); the local committee responsible for Germany – the Medical Faculty of the Friedrich-Alexander University in Erlangen-Nürnberg (Approval No. 119_14Mz); and the local ethics committee responsible for Northwestern and Central Switzerland (Ethikkommission Nordwest und Zentralschweiz EKNZ) (Approval No. EKNZ 2014–106).

### Interventions

All study interventions are described in Table 2 using the Template for Intervention Description and Replication checklist and guide, known as TIDieR. Patients will be evenly allocated to CaCT, AP, or ACT. All groups will additionally receive conventional neurorehabilitative treatment as prescribed by each clinic. The experimental intervention of this trial will be added to the existing therapy plan as a daily treatment of 30 minutes for 4 weeks (Monday to Friday). Every treatment will be documented on the therapy documentation sheet (standardised for each trial centre).

**Table 2:** Description of study interventions based on the TIDieR template^*a^

Item	Computer-assisted cognitive training	Acupuncture	ACoTrain
1 Short name	Ca CT	AP	ACT
2 Why	Main objective is to evaluate the effects of computer-assisted cognitive training CaCT compared to AP on the outcomes of attention assessments		
	Second objective is to test the effects of a combined cognitive intervention that incorporates CaCT and AP		
3 What: materials	Ca CT (RehaCom, Hasomed Inc., Germany, http://www.hasomed.de) will be conducted in the study. Patients will sit in front of the RehaCom system. The training program will be displayed on the computer screen and they also can hear the sound feedback from the audio system. According to different training programs, patients will press a special RehaCom keyboard with large buttons to react	Acupuncture needle, which is single use, φ 0.35 × 40 mm, will be used for acupuncture treatment. The angle of insertion is approximately 10°–20° (between needle and scalp), the needle should be inserted to a depth of approximately 0.3–0.5B-cun	For the combined group, patients will receive AP and CaCT combined, i.e. the CaCT will be performed while the needles are inserted
4 What: procedures	For the purpose of this study, three submodalities of attention will be targeted with CaCT: intensity, selectivity, and divided attention [26]. To approach intensity of attention, the RehaCom modules “Attention and Concentration (AUFM)” and “Vigilance (VIGI)” will be used. For selectivity, the RehaCom task called “Alertness” for reaction behaviour and reactivity will be used. Divided attention will use the task called “Divided Attention Training 1 and 2”. Each task will start at the lowest difficulty level to allow progression according to the patient’s abilities. Only “Attention and Concentration” and “Divided Attention Training 2” will begin with level 4 and 2 respectively. A detailed description of the tasks and their content can be found on the developer’s site (Hasomed GmbH, www.hasomed.de)	The treatment will be performed after sterilisation of the skin on the areas where the needles will be inserted. The patient will be instructed to lie supine or to sit down on a treatment chair while the doctor’s left hand fixates the target position (DU20 and DU24), The angle of insertion is approximately 10°–20°(between needle and scalp), the needle should be inserted to a depth of approximately 0.3–0.5B-cun. Following insertion, a 1-minute stimulation of the acupuncture point will be performed using bidirectional rotation of the needle’s sleeve. This provokes a sensation known as Deqi, which is commonly described as a ‘glowing’ feeling. The needle will be kept in this position for 30 minutes. The described manipulation will be repeated every 10 minutes and the needles will be withdrawn after the fourth manipulation	
5 Who provides	RehaCom training will be provided by experienced neuropsychology therapists, who will have at least 2 years of professional experience in the field of neurorehabilitation.	Acupuncture will be performed by acupuncturists who have a TCM practitioner’s license, and at least 2 years of working experience	Both study interventions will be provided by experienced neuropsychology therapists and acupuncture therapists, who will have at least 2 years of professional experience
6 How	All the study interventions will be conducted individually in one-to-one sessions		
7 Where	All the study interventions will take place in the neuropsychology therapy department of each participating centre		
8 When and how much	During the 4-week intervention program, patients in each study group will receive the same amount of 20 sessions lasting 30 minutes each		
9 Tailoring	Training and therapy content will be tailored to each patient’s preferences, and the concentration level of each patient		

^*^ *Ca CT* computer-assisted cognitive training, *AP* acupuncture, *ACT* AcoTrain, *TCM* Traditional Chinese Medicine

^a^Items 10, 11 and 12 of the Template for Intervention Description and Replication checklist and guide (TIDieR) template do not apply to this study

### Conventional therapy

All participants will receive basic treatment according to the rehabilitation concept applied in the individual clinics and/or as demanded by cost bearers. Multidisciplinary treatment will include physiotherapy, occupational therapy, medical exercise therapy, hydrotherapy, etc. Patients will not be restrained from CaCT or AP training if this is part of the conventional therapy of the individual clinic. However, each trial centre is instructed to track the types of interventions provided and provide precise documentation of therapy treatments, medication, therapy frequency and dose.

### Acupuncture treatment

For every treatment visit, participants are treated with two acupuncture points [Baihui (GV20)] and [Shenting (GV24)]. Patients will be treated by an experienced acupuncturist with a working experience of at least 2 years in neurological rehabilitation with acupuncture. The targeted acupoints are described in detail in Table 3.

**Table 3:** Acupuncture points selected in this protocol

Acupuncture points	Location
Baihui (GV20)	On the head, 5 ^*^B-cun superior to the anterior hairline, on the anterior median line
	Note 1: GV20 is located in the depression 1 ^*^B-cun anterior to the midpoint of the line from the anterior hairline to the posterior hairline
	Note 2: when the ears are folded, GV20 is located at the midpoint of the connecting line between the auricular apices
Shenting (GV24)	On the head, 0.5 ^*^B-cun superior to the anterior hairline, on the anterior median line.

^*^B-cun: proportional bone cun. This method divides the height of the human body into 75 equal units. Using joints on the surface of the body as the primary landmarks, the length and width of every body part is measured by such proportions

Before treatments, the targeted acupoints are sterilised with alcohol. While one hand fixates the target position, the other hand is used to insert the needle (Huatuo, single use, φ0.35 × 40 mm, distributor Suzhou HuaTuo Medical Instruments, Suzhou, China). The angle of insertion is approximately 10°–20° (between needle and scalp), the needle should be inserted to a depth of approximately 0.3–0.5 cun (1 cun = 3.33 cm). Following insertion, a 1-minute stimulation of the acupuncture point will be performed using bidirectional rotation of the needle’s sleeve. This provokes a sensation known as Deqi, which is commonly described as a ‘glowing’ feeling. The needle will be kept in this position for 30 minutes. The described manipulation will be repeated every 10 minutes and the needles are withdrawn after the fourth manipulation.

### Computer-assisted cognitive training with RehaCom

It is important to note that the CaCT group will use RehaCom (Hasomed Inc., Magdeburg, Germany, http://www.hasomed.de) in our study. Three rehabilitation centres from different countries will be involved in our study. Traditional cognitive training programs are delivered in individual or group format by a trained instructor, and differ primarily with regards to trained abilities, length and frequency of training, and specific strategies practiced [22]. Therefore, it is better to use a computer-assisted training program to ensure patients from different centres will all receive equal treatments. RehaCom has been translated into different languages and extensively used in cognitive rehabilitation following the relevant diseases, for example, stroke [23], schizophrenia and multiple sclerosis [24, 25].

The software has five different therapeutic programs aimed at restoration of attention, memory, executive functions, or visual field. Each program has one to four different tasks to choose from for each therapy session.

For the purpose of this study, three submodalities of attention will be targeted with CaCT: intensity, selectivity, and divided attention [26]. To approach intensity of attention, the RehaCom task called “Attention and Concentration” and “Vigilance” will be used. For selectivity the RehaCom task called “Alertness” for reaction behaviour and reactivity will be used. Divided attention will use the task called “Divided Attention Training 1 and 2”. Each task will start at the lowest difficulty level to allow progression according to the patient’s abilities. Only “Attention and Concentration” and “Divided Attention Training 2” will begin with level 4 and 2 respectively. A detailed description of the tasks and their content can be found on the developer’s site (Hasomed GmbH, www.hasomed.de).

### ACoTrain: acupuncture and computer-assisted attention training

For the combined group, patients will receive AP and CaCT combined, i.e. the CaCT will be performed while the needles are inserted.

### Outcome assessment

Outcomes will be measured once before intervention (T0) and once after intervention (T1, at 4 weeks) in a 1- to 1.5-hour assessment. Assessments will be conducted by a trained and experienced therapist or neuropsychologist blinded to group allocation. None of the assessors will participate in the treatment. The study will use a number of standardised scales to assess functional changes. At baseline, additional assessment of basic cognitive functioning will be administered for classification of the included patients.

### Primary outcome measure

#### Test for Attentional Performance (TAP)

The Test for Attentional Performance (TAP) is a computer-assisted standardised neuropsychological test [27] used to evaluate attention deficits. The procedures included in the test battery are: Alertness and Go/No Go for basic attention and general anticipation, incompatibility for divided visual-spatial attention, and divided attention. Outcomes are measured in reaction time (ms) and reported as percentile ranks. The TAP test has previously been shown to be both reliable and valid [28, 29]. Alertness is required for effective behaviour and deemed the basis of every attention performance. It is tested under two conditions measuring reactions times.

- React to a randomly appearing cross on the screen (click a button)
- React to a critical stimulus preceded by a cue stimulus presented as warning tone
  Go/No-Go task tests the ability to suppress an inappropriate reaction (control of impulsive behaviour). This is achieved by measuring the amount of correct reaction to the target stimuli. Five different patterns of a square are presented, only two are correct
  Incompatibility, a right- or left-pointing arrow appears on the left or right side of the screen. The patients are instructed to indicate the direction of the arrow, not the location of the arrow
  Divided attention assesses the capacity to pay attention to simultaneously ongoing processes. The test presents a visual and an auditory task, which must be processed in parallel (dual task)

### Secondary outcome measures

#### Trail Making Test (TMT)

The TMT is a popular neuropsychological test and is included in most cognitive test batteries [30]. The TMT provides information on visual search, scanning, speed of processing, mental flexibility, and executive functions. The TMT consists of two parts. TMT-A requires an individual to draw lines sequentially connecting 25 encircled numbers distributed on a sheet of paper. Task requirements are similar for TMT-B except the person must alternate between numbers and letters (e.g. 1, A, 2, B, 3, C, etc.). The score on each part represents the amount of time required to complete the task(s). The Chinese version of the TMT-B uses Digit Symbol Coding [31]. The reliability and validity of the TMT test has previously been shown [32, 33].

#### Test des Deux Barrages (T2B)

The T2B is a commonly applied assessment in clinical neuropsychology to test selective attention. The original version consists of two parts, one time-independent, the second limited to 10 minutes. For the purpose of this study, only the latter will be applied. The test is presented on a large A3 sheet that displays two symbols in the header line and a 40 × 25 matrix with a total of 1000 randomly assorted symbols similar to those in the header line. Only a few of the matrix symbols are identical to the header symbols. The participant is instructed to mark only identical ones. Test duration is 10 minutes. After every minute, the assessor notes the progress of the participant indicating the line and column. After 10 minutes, the amount of falsely marked symbols is counted using a template solution. The task is to work as quick and accurately as possible. The parameters to quantify this are: time needed (if less than 10 minutes), total amount of correct markings per minute, amount of omitted symbols per minute, amount of wrong markings per minute, sum of errors per minute (omitted or wrong marking), percentage of errors, and range of performance (highest frequency of correct markings per minute to lowest frequency of correct markings per minute).

#### National Institute of Health Stroke Scale (NIH-SS)

The NIH-SS is a standardised assessment of neurological functions in the acute phase of stroke [34]. It is generally used to quantify improvement of the patient’s neurological impairments on 15 items in 11 areas of different neurological status. The administering medical doctor observes the patient and rates each item on an ordinal scale with three to five levels. A maximum of 42 points can be achieved, which would correspond to severe neurological impairment. The NIH-SS will be recorded once to allow classification and description of the severity of stroke of the included patients. It is generally provided by the referring hospital. If not, the treating neurologist may reconstruct it retrospectively [35].

#### Modified Barthel Index (MBI)

The MBI assesses impairment of daily activities in patients with neurological disorders and takes into account available support (personal or device-specific) [36]. The scale rates motor and cognitive functions on a five-point Likert scale (0–4, the higher the less dependent).

#### EuroQol Questionnaire (EQ-5D)

The EQ-5D is a generic self-report instrument for use as a measure of health outcome [37]. Applicable to stroke, but also other health conditions [38], it provides a simple descriptive profile and a single index value for health status.

### Data collection methods and management

Data will be collected on digitalised Case Report Forms (e-CRF) that will resemble the paper forms approved by the ethics committee (p-CRF). Data will be transferred from the output files and assessment forms to the e-CRFs and will not be part of the patient’s case history. Forthwith, only a participant ID will be connected to the data. The CRF will be transferred to the data administrator of the Data Control Centre (DCC, party A) after being signed (digitally) by the clinical inspector. The DCC will verify consistency, completeness, and accuracy of submitted data forms. All participating trial centres will be contacted by the DCC in case of uncertainties regarding the submitted data and the DCC will inform all trial centres on the status of the database.

All data is treated with utmost confidentiality and made anonymous to anyone outside the study (except the ethics committees during audit). Any further analyses will only refer to ID numbers. The key will only be accessible to study personnel with a password for the protected local drive where data is saved. All data and related documents remain archived for 10 years at each study centre before being shredded. All trial centres will receive anonymous copies of all data in order to promote dissemination of study results.

Entering data forms and general information about obtaining data was covered during the training session prior to study commencement.

### Statistical methods

All allocated subjects will be analysed with the available data, i.e. on the basis of intention-to-treat (ITT). The last observation carried forward method will be applied in case of missing data.

A significance level of 95 % (two sided *p* values alpha <0.05) will be used. After examining the data for normality (the Kolmogorov-Smirnov test) the outcome measures will be analysed using ANOVA (assuming normal distribution) or Kruskal-Wallis (nonparametric test). Demographic characteristics and other baseline values will be described using descriptive statistics for each group. Significant group interactions will be analysed post hoc. The Bonferroni method will be used to appropriately adjust the overall level of significance for multiple outcomes. A statistician blinded to study groups will conduct statistical analysis.

### Patient safety

Adverse effects attributable to the RehaCom therapy may include mental fatigue or headache. Symptoms usually resolve in patients as they progress with therapy and become more familiar with it [39]. Acupuncture may cause discomfort or bruising at the sites of needle insertion, nausea, or feeling faint after each treatment.

Any medical occurrence after inclusion, which is not necessarily caused by the intervention (adverse events, AEs) will be recorded on the individual CRFs and on a progress protocol. This report will be sent to the ethics committee and the sponsor once a year as a safety report.

Serious adverse events (SAEs) will be reported to the sponsor immediately and all participating clinics will be informed by the sponsor. It is the responsibility of each study centre to inform the local ethics committee within 7 days.

### Quality control

To ensure that treatments are of a high standard and delivered in accordance with the trial protocol, clinicians involved in assessments, treatment or training will have to provide a proven record of at least 3 years of clinical experience and certified training or education in related fields of rehabilitation or research. The acupuncture will only be performed by professional physicians with specific training in TCM and a minimum of 2 years of experience. They will participate in a 3-day training course in the standard operating procedures (SOPs) provided by the author of the mutualised protocol and the standard operation videos. In this training course, the protocol will be explained and practiced during exercises and role plays.

## Discussion

Cognitive impairments, such as, memory, executive function, and attention deficit frequently occur in stroke patients, though sometimes, attentional deficit is the only cognitive impairment apparent [40]. Sustaining attention is a prerequisite of motor relearning [41], which is essentially the mechanism of functional recovery after stroke [42]. The traditional neurocognitive strategies [43] used to improve attention deficit after stroke often result in incomplete resolution, and thus, more effective adjuvant strategies are needed.

Acupuncture focuses on the global cognitive function from the holistic conception of TCM, while the RehaCom cognitive training concerns the specific impaired cognitive functions, which need to be trained from the viewpoint of symptomatic treatment. However, the cognitive impairment after stroke is so complex that it needs an integrated method.

The aims of our study are to compare CaCT and AP efficacy in terms of attention improvement after stroke and to investigate the potential effect of a combination of both (ACT). The participants will have the study treatment added to their existing rehabilitation program and will be assessed for changes in attention (intensity, selectivity, and divided attention) at 4 weeks.

Although CaCT and AP both have shown to have beneficial effects on overall cognition in various studies, they have not been compared directly and for specific attentional performance. This is the first study of its kind that assesses attention-specific outcomes for both methods and not only compares the methods but also advances into new approaches where two methods well accepted in two very different cultures are combined to complement one another. The multicentric approach allows more generalisable interpretation [44] of the results and the usage of standardised and cross-culturally adapted assessments allows comparison and pooling of all study data coming from different trial centres.

Given its small sample size, the pilot study will mainly produce results to provide estimation of large-scale study sample sizes and test the feasibility of the international multicentric study design and its management. A further limitation of the study is the distinction of effects caused by the conventional training as opposed to the experimental interventions. However, the primary aim of the study is a parallel comparison of three experimental interventions rather than comparison to conventional therapy. Hence, no control group receiving no experimental intervention was included for methodological and ethical reasons. This allowed the same amount of time spent with a therapist in all intervention groups. That is deemed as an important measure to prevent bias, particularly when patients cannot be blinded to interventions, as it exposes each group to the same amount of potential placebo effects [45].

The ACoTrain study intends to assess stroke patients of very different cultures (Asia and Europe). Great care has been taken to select cross-culturally adapted and validated assessments to allow pooling of the final data. The assessments are designed to measure the same construct, independent of cultural, educational, or lingual differences. Further, patients in early stroke rehabilitation often present with impairments in other areas than cognition. When assessing cognition, however, these impairments may confound the outcomes, as the task at hand cannot be isolated from other motor and cognition functions. Effects of motor disabilities, impaired perception (e.g. visual deficits, reduced proprioception), or other cognitive defects (e.g. memory) and lack of motivation can limit the interpretability of attention assessments [46]. The eligibility criteria were carefully selected to exclude such confounding factors. To further promote internal validity of the assessments, trained and experienced neuropsychologists will educate all assessors accordingly. To achieve this, a series of assessment training workshops for all personnel involved in the study have been held twice prior to the beginning of the study (March 2014 and August 2014 in Germany and China respectively).

## Trial status

Ongoing recruitment.

## Abbreviations

ACoTrain: ACT, intervention that incorporates CCT and AP
AEs: Adverse events
AP: Acupuncture
BL: Baseline
Ca CT: Computer-assisted cognitive training
DCC: Data Control Centre
e-CRF: Digitalised Case Report Form
EQ-5D: EuroQol Questionnaire
ITT: Intention-to-treat
KAP: Short aphasia test
MBI: Modified Barthel Index
MoCA: Montreal Cognitive Assessment
NIH-SS: National Institute of Health Stroke Scale
p-CRF: Paper Case Report Form
RCTs: Randomised controlled trials
SAEs: Serious adverse events
SOPs: Standard operating procedures
T2B: Test des Deux Barrages
TAP: Test for Attentional Performance
TCM: Traditional Chinese Medicine
TIDieR: Template for Intervention Description and Replication checklist and guide
TMT: Trail Making Test
